# Dynamics of natural prokaryotes, viruses, and heterotrophic nanoflagellates in alpine karstic groundwater

**DOI:** 10.1002/mbo3.98

**Published:** 2013-07-04

**Authors:** Inés C Wilhartitz, Alexander K T Kirschner, Corina P D Brussaard, Ulrike R Fischer, Claudia Wieltschnig, Hermann Stadler, Andreas H Farnleitner

**Affiliations:** 1 Department of Environmental Microbiology, Eawag, Swiss Federal Institute of Aquatic Science and TechnologyÜberlandstrasse 133, 8600, Dübendorf, Switzerland; 2 Institute for Hygiene and Applied ImmunologyWater Hygiene, Medical University of Vienna1090, Vienna, Austria; 3 Interuniversity Cooperation Center Water and HealthGumpendorferstr. 1a, 1060, Vienna, Austria; 4 Department of Biological Oceanography, Royal Netherlands Institute for Sea Research (NIOZ)1790 AB, Den Burg, Texel, The Netherlands; 5 Aquatic MicrobiologyInstitute for Biodiversity and Ecosystem Dynamics, University of AmsterdamAmsterdam, The Netherlands; 6 Joanneum Research, Institute for Water, Energy and Sustainability, Research Group Water Resources ManagementElisabethstrasse 18/II, 8010, Graz, Austria; 7 Research Group Environmental Microbiology and Molecular Ecology, Institute of Chemical EngineeringGumpendorferstr. 1A, 1060, Vienna, Austria

**Keywords:** Alpine karst, groundwater, heterotrophic nanoflagellates, prokaryotes, viruses

## Abstract

Seasonal dynamics of naturally occurring prokaryotes, viruses, and heterotrophic nanoflagellates in two hydro-geologically contrasting alpine karst springs were monitored over three annual cycles. To our knowledge, this study is the first to shed light on the occurrence and possible interrelationships between these three groups in karstic groundwater. Hydrological and microbiological standard indicators were recovered simultaneously in order to estimate surface influence, especially during rainfall events. Data revealed a strong dependence of the microbial communities on the prevailing hydrological situation. Prokaryotic numbers averaged 5.1 × 10^7^ and 1.3 × 10^7^ cells L^−1^, and heterotrophic nanoflagellate abundance averaged 1.1 × 10^4^ and 3 × 10^3^ cells L^−1^ in the limestone spring type (LKAS2) and the dolomitic spring type (DKAS1), respectively. Viral abundance in LKAS2 and DKAS1 averaged 9.4 × 10^8^ and 1.1 × 10^8^ viruses L^−1^. Unlike in DKAS1, the dynamic spring type LKAS2 revealed a clear difference between base flow and high discharge conditions. The virus-to-prokaryotes ratio was generally lower by a factor of 2–3, at higher average water residence times. Furthermore, the high prokaryotes-to-heterotrophic nanoflagellate ratios, namely about 4700 and 5400 for LKAS2 and DKAS1, respectively, pointed toward an uncoupling of these two groups in the planktonic fraction of alpine karstic aquifers.

Seasonal dynamics of naturally occurring prokaryotes, viruses and heterotrophic nanoflagellates in two hydro-geologically contrasting alpine karst springs were monitored over three annual cycles. Data revealed a strong dependence of the microbial communities on the prevailing hydrological situation.

## Introduction

It is in karsts that the roots of the idea of an groundwater ecosystem are found (Rouch 1977). About 20% of the earth's surface can be defined as karst landforms and the related aquifers are unique in terms of hydrogeology, biogeochemistry, and vulnerability compared with other groundwater ecosystems (Ford and Williams 1996; Drew and Hötzl 1999). But although 25% of the global population is partly or completely supplied by water from such areas (Ford and Williams 2007), still only limited information about microbial community structures, dynamics, and related microbial activity is available in fractured media aquifers, which is especially true for karst aquifers and their respective spring waters (Cho and Kim 2000; Lehman et al. 2001a,b; Simon et al. 2001; Roudnew et al. 2013). Studies on the entire naturally occurring prokaryotes, virioplankton community, or protozoans are sparse (Williamson et al. 2002; Farnleitner et al. 2005; Goddard et al. 2005) as the main focus is mostly toward health relevant organisms, for example, bacteriophages infecting enteric bacteria (Gordon and Toze 2003; John and Rose 2005; Lucena et al. 2006). Data are needed to gain better insights into the true importance of these compartments in nutrient cycles and groundwater quality.

In alpine groundwater systems, especially those with high average water residence times, it can be assumed that important pathways of energy and carbon flux are through the microbial loop. Therein, prokaryotes play a crucial role as they consume and recycle dissolved organic carbon (DOC) and viruses can have a profound influence on geochemical cycling (Säwström et al. 2006; Suttle 2007; Brussaard et al. 2008) also affecting the cycle of nutrients such as nitrogen and phosphorus (Wilhelm and Suttle 1999).

Recently, first evidence was given for the presence of aquifer specific autochthonous microbial endokarst communities (AMEC) in alpine karst aquifers by our research group (Farnleitner et al. 2005). These findings were since then confirmed by other studies in karstic aquifers (Pronk et al. 2006, 2009a; Wilhartitz et al. 2009). Thus, on the search for biotic factors possibly controlling the prokaryotic compartment we investigated viral and protozoan abundance in two different spring types. The focus of this study was on the planktonic fraction, as (i) there are established means to discriminate between surface influenced waters (external factors) and the base flow component (Farnleitner et al. 2005; Wilhartitz et al. 2009), (ii) there is an actual interest for water authorities in terms of biostability of these water resources, and (iii) we wanted to find out to which extent karstic groundwater differs from other aquatic environments. The latter relates to the feature that aquifer water is in perpetual contact with biofilms on bedrock and sediments, that constitute a different habitat than the water itself in terms of cell numbers and turnover rates, which are orders of magnitude higher in the biofilm (Wilhartitz et al. 2009).

In order to capture the fluctuation margin of possible prevailing conditions in karstic aquifers two study sites were chosen that – by their nature – can be allocated at both ends of a spectrum covering different hydrographical conditions representative for the central European region. A very dynamic and large limestone spring type (LKAS2) and a conservative dolomitic one with a long average water residence time (DKAS1). Thus, these two springs offer unique systems to study the interdependence between prokaryotic, viral, and heterotrophic nanoflagellates (HNF) abundance. It can be supposed that nutrients in DKAS1 are partly derived from a turnover of autochthonous sources, whereas in LKAS2 there is also considerable allochthonous nutrient supply during some time of the year (Wilhartitz et al. 2009).

To our knowledge, this is the first time study reporting on long-term investigation data of prokaryotic, viral, and HNF abundances in the water column of two ultraoligotrophic alpine karst aquifers. The aim was to elucidate the occurrence and dynamics of three microbial groups and to investigate their dependence on the given hydrological situation. Possible systemic differences between the two different aquifer types were elaborated.

## Material and Methods

### Study site

The selected ground waters from two different alpine springs (LKAS2 and DKAS1) are located in the Northern Calcareous Alps in Austria (detailed description in Farnleitner et al. 2005). They have nearby catchment areas but differ in terms of their hydrogeological characteristics. LKAS2 represents a typical limestone spring type (D'Amore et al. 1983), with an average discharge of 5200 L sec^−1^, showing pronounced discharge fluctuations throughout the seasonal cycle. During flood events discharge can rise up to 25,000 L sec^−1^ including surface runoff which implies considerable surface input (allochthonous nutrients and microorganisms) into the aquifer (Stadler et al. 1998; Reischer et al. 2006, 2007). DKAS1 on the other hand, represents a typical dolomitic-limestone spring type (D'Amore et al. 1983) with an average discharge of 300 L sec^−1^, rather constant discharge conditions and an average water residence time of about 22 years (Stadler and Strobl 1997).

### Sampling

Samples (total volume of 4.2 L) were collected from October 2002 to December 2005 every 3–4 weeks directly at the spring outlet. The water was transported at in situ temperature, divided into duplicates for prokaryotes, viruses, and HNF, respectively, and fixed (formaldehyde, final concentration 2%) within 2–3 h. Subsamples were stored at 4°C. Additional subsamples for enumeration of prokaryotes and viruses with the flow cytometer (FCM) were collected for one annual cycle (August 2004 to August 2005) and duplicates were fixed and frozen in liquid N_2_ directly at the spring outlet (Brussaard 2004).

The most evident problem when studying AMEC in the spring effluent is that it is not possible to discriminate between planktonic fractions and cells that might have been released from the biofilm compartment. Still, as there are no other access points to the groundwater reservoir, sampling the spring outlet is the only possibility to obtain in situ data of the microbial communities inhabiting this ecosystem.

### Hydrological parameters

All hydrological and chemo-physical data were recovered by in-field on-line sensors. Conductivity, water temperature, and discharge related parameters (water pressure, current meters, inductive discharge measurements) were registered with the data collecting system GEALOG-S from Logotronic (Vienna, Austria). The conductivity and pressure probes were from WTW-Tetracon 96A (WTW, Weilheim, Germany) and PDCR 1830 (Druck, London, UK). Impeller flow sensors were Peek 400 models (Peek, Houston, TX) and ELIN Water Technology (Vienna, Austria) current meters were used. Signals from these sensors were converted with algorithms provided by the manufacturers and from discharge stage relations (Stadler et al. 1998). Data were stored every 15 min. Turbidity, SAC_254_ (spectral absorbance coefficient at 254 nm) and pH were measured with s::can sensors with integrated data logger (s::can, Vienna, Austria). Turbidity and SAC_254_ were not measured in DKAS1 as former observations showed that these parameters were constant, due to the nature of the aquifer (Stadler et al. 1998). Conductivity measurements in this study confirmed the steady hydrological conditions within this aquifer during the investigation period.

### Standard microbiological water quality parameters

Enumeration of *Escherichia coli* and heterotrophic plate counts (HPC), incubated at 22°C (HPC22) was performed as described elsewhere in detail (Farnleitner et al. 2010).

### Prokaryote abundance, morphotypes, and biomass

All samples for biological measurements were collected in duplicates. For examination by epifluorescence microscopy (EFM), a slightly modified version of the acridine orange direct count method after Hobbie et al. (1977) was applied. Filters were examined under a Leitz Diaplan epifluorescence microscope (Leica, Wetzlar, Germany) equipped with a HBO 50 W mercury lamp (excitation wavelength 450–490 nm, cutoff filter 515 nm). Prokaryotic cells were separated into three classes according to their different morphology: rods, cocci, and vibrios (curved rods). Other forms were of negligible importance. At least 10 microscopic fields per sample were counted and the length and width of 100–150 cells was measured (>30 per morphotype). To estimate the average prokaryotic biomass (PB), an average cellular carbon content of 15 and 12 fg C per cell was used for LKAS2 and DKAS1, respectively (Wilhartitz et al. 2009).

In addition to EFM, FCM was applied during one seasonal cycle to examine the counting efficiency of EFM, as it was sometimes difficult to distinguish between prokaryotes, viruses, and small particles during high water events. Subsamples for virus and prokaryote enumeration were prepared as described earlier (Marie et al. 1999; Brussaard 2004). In short, samples were fixed with glutaraldehyde (0.5% final concentration, EM-grade; Merck, Darmstadt, Germany) for 30 min at 4°C, followed by freezing in liquid nitrogen and storage at −80°C. Samples for prokaryotes were diluted (10× to 100×) in TE buffer (10 mmol L^−1^ Tris-HCl and 1 mmol L^−1^ EDTA, pH 8) and stained with SYBR Green I (final concentration 1 × 10^−4^ dilution of commercial stock; Invitrogen Inc., Eugene, OR) for 10 min. A FACSCalibur flow cytometer (Becton Dickinson, San Jose, CA) equipped with a 15-mW 488-nm air-cooled argon laser and a standard filter setup was used. The trigger was set on green fluorescence. Total counts were corrected for the blank obtained with TE buffer only.

### Viral abundance and biomass

For EFM analysis 1 to 2 mL sample was filtered through a 0.02 μm-pore-size Al_2_O_3_ Anodisc membrane filter (Whatman International, Maidstone, U.K.) backed by a 0.2-μm pore size cellulose nitrate filter (Sartorius, Göttingen, Germany) at ~20 kPa vacuum. After staining with SYBR Gold (Molecular Probes, Eugene, OR; 2.5 × 10^−3^ final dilution of the stock solution) for 15 min in the dark, the filters were dried and mounted on a glass slide with a drop of 0.1% *p*-phenylenediamine (made freshly from a frozen 10% aqueous stock; Sigma Aldrich, Munich, Germany) in Citifluor (Glycerol/PBS solution-AF1; Agar Scientific Ltd, Stansted, U.K.) (Chen et al. 2001; Noble 2001; Fischer et al. 2005). Filters were examined at a magnification of 1250× as described for prokaryotes. To estimate the concentration of viruses, at least 200 viruses per subsample were counted. Biomass was calculated using a conversion factor 0.2 fg C phage^−1^ (Suttle 2005).

Samples for FCM (Becton Dickinson) were treated as described for prokaryotes (see above) except that viruses were stained at 80°C for 10 min and allowed to cool down for 5 min prior to analysis (SYBR Green I, final concentration 5 × 10^−5^ dilution of commercial stock; Invitrogen Inc.) (Brussaard 2004).

### HNF abundance and biomass

Fifty milliliters of spring water was stained with 0.5 mL of 4′,6-diamidino-2-phenylindole (final concentration 1 μg mL^−1^). After 30 min, the mixture was filtered through a black 0.8-μm pore size polycarbonate filter (Millipore, Vienna, Austria) backed by a 0.45-μm pore size cellulose nitrate support filter (Sartorius) (Wieltschnig et al. 2003). The filter was washed three times with 1 mL formaldehyde (1% solution) to remove excess dye, air dried on a microscopic slide, mounted in a drop of paraffin-oil, covered with a cover slip, and stored at −20°C for a minimum of 2 h and a maximum of 2 weeks. Filters were examined by EFM (Wieltschnig et al. 2001). Duplicate filters per sample were counted. The detection limit was 800 HNF L^−1^, taking into account the precision of the counts and the time needed for the inspection of one filter. Biovolumes were estimated assuming cells to have standard geometrical shapes and were converted to carbon biomass assuming a conversion factor of 0.22 pg μm^−3^ (Borsheim and Bratbak 1987; Wieltschnig et al. 2003).

### Statistics

For statistical analysis the software package IBM SPSS Statistics 19 was used (IBM, Zürich, Switzerland). The Mann–Whitney rank test was applied for comparison of two groups of values. To compare multiple groups, one-way analysis of variance (ANOVA) was used. Differences were considered to be significant at a confidence level of *P* < 0.05 in all tests.

## Results

### Hydrological conditions and basic spring water quality characteristics

The dolomitic spring type DKAS1, showing high average water residence time, displayed a rather constant discharge (*Q*) throughout the study, with a mean discharge of 303 L sec^−1^ (*Q*_max_/*Q*_min_ discharge ratio of ~1/1.45) whereas the limestone spring type LKAS2 showed a mean discharge of 4188 L sec^−1^ and strong seasonal variations (*Q*_max_/*Q*_min_ discharge ratio of ~1/42) (Table 1). High correlations between discharge and water quality characteristics, such as conductivity, turbidity, and SAC_254_, were observed in LKAS2 (Table 2A). The strong influence on water quality by the prevailing hydrological conditions in LKAS2 was confirmed by fluctuation of HPC22 and *E. coli,* two parameters routinely used in water analysis. HPC22 varied up to three orders of magnitude (Table 1) with numbers being highest during flood events. Numbers of *E. coli* also varied significantly, but detection was limited to the warmer season and increased discharge.

**Table:** Hydrogeographical and microbial characterization of two different alpine karst spring waters

Parameter^1^	Unit	LKAS2				DKAS1					
		Median	Range min–max	Median	Range min–max
Q	(L sec^−1^)	4188	902–15,479	303	241–373
EC	(μS cm^−1^)	200.7	156.2–224.0	339.0	333.0–354.9
SAC	(m^−1^)	1.40	0.44–3.89	n.d.	n.d.
TUR	(NTU)	0.19	0.03–0.96	n.d.	n.d.
TEMP	(°C)	5.50	4.09–5.95	6.67	6.61–6.77
HPC22^2^	(CFU/mL)	31.5	0–420	0	0–3^3^
*E. coli*	(CFU/100 mL)	2.5	0–230	0	0–10^3^
PN	(10^6^ cells L^−1^)	50.6	25.6–84.9	13.2	8.0–19.0
PN-R	(10^6^ cells L^−1^)	30.6	25.6–84.9	6.5	4.0–9.6
PN-C	(10^6^ cells L^−1^)	14.8	6.8–30.7	6.1	3.6–10.9
PN-V	(10^6^ cells L^−1^)	3.2	0.5–7.7	0.5	0.2–2.0
VN	(10^7^ viruses L^−1^)	94.4	5.5–317	10.9	1.1–37.6
HNF	(10^6^ cells L^−1^)	10.9	3.3–50.7	3.3	0.8–12.8
PB	(nmol C L^−1^)	63.2	32.0–106.1	13.8	8.0–16.3
VB	(nmol C L^−1^)	15.7	0.9–52.8	1.8	0.2–6.3
HNF-B	(nmol C L^−1^)	8.9	2.7–41.7	2.7	0.7–10.5
TB	(nmol C L^−1^)	87.8	35.6–200.6	18.3	8.9–33.1

*Q*, discharge; EC, electrical conductivity; SAC, spectral absorbance coefficient at 254 nm; TUR, turbidity; NTU, nephalometric turbidity unite; TEMP, temperature; HPC, heterotrophic plate count; *E. coli*,* Escherichia coli* abundance according to ISO 9308-1; PN, prokaryotic number; PN-R, rod-shaped PN; PN-C, coccus-shaped PN; PN-V, vibrio-shaped PN; VN, viral abundance by epifluoresence microscopy; HNF, heterotrophic nanoflagellate abundance; PB, prokaryotic biomass; VB, viral biomass; HNF-B, heterotrophic nanoflagellates biomass; TB, total biomass; n.d., not detected (proven to be stable, see Material and Methods).

1 Samples were taken monthly over a 3-year-period (*n* = 40).

2 HPC at 22°C.

3 These parameters were only detected twice throughout the study.

**Table:** Spearman coefficients for LKAS2 (A) and DKAS1 (B)

A										
	*Q*	EC	SAC	TURB	TEMP	HPC	*E. coli*	PN	PN-R	VN
EC	−0.74^1^									
SAC	0.72^1^	−0.42								
TURB	0.61	−0.27	0.32							
TEMP	−0.56	0.91#mbo^1^	−0.31	−0.09						
HPC	0.65	−0.72^1^	0.67^1^	0.42	−0.65^1^					
*Ecoli*	0.41	−0.66^1^	0.37	0.14	−0.77^1^	0.71^1^				
PN	0.68^1^	−0.55	0.56	0.54	−0.43	0.69^1^	0.51			
PN-R	0.67^1^	−0.58	0.74^1^	0.40	−0.53	0.74^1^	0.68^2^	0.70^1^		
VN	0.79^1^	−0.77^1^	0.61	0.36	−0.72^1^	0.72#mbo^1^	0.70#mbo^1^	0.57	0.68^1^	
HNF	0.56	−0.64^1^	0.39	0.55	−0.63^1^	0.57	0.49	0.61^2^	0.45	0.67^1^
B					
	*Q*	EC	TEMP	PN	VN
EC	−0.64^1^				
TEMP	−0.18	−0.058			
PN	0.33	−0.42	−0.21		
VN	0.31	−0.34	0.28	0.10	
HNF	−0.29	0.29	0.28	−0.17	0.33

*Q*, discharge; EC, electrical conductivity; SAC, spectral absorbance coefficient at 254 nm; TUR, turbidity; TEMP, temperature; HPC, heterotrophic plate count; *E. coli*, *Escherichia coli* abundance according to ISO 9308-1; PN, prokaryotic number; PN-R, rod-shaped PN; VN, viral abundance by epifluoresence microscopy; HNF, heterotrophic nanoflagellate abundance (proven to be stable, see Material and Methods). Bonferroni corrected.

1 Significance at the 0.01 level is marked.

2 Significance at the 0.05 level (*n* = 40).

The difference between sites for prokaryotic, viral, and HNF abundance was significant (one-way ANOVA, *n* = 80 for each set, *P* < 0.0005), especially when comparing the influence of winter and summer seasons. In DKAS1 the distribution of all three microbiological groups was the same throughout the year, meaning that the null hypothesis, “the distribution is the same across categories of seasons”, could be retained (Mann–Whitney *U*-test; prokaryotes *P* < 0.463, virus *P* < 0.465, HNF *P* < 0.755; *n* = 34). In LKAS2 the null hypothesis had to be rejected for all three groups (Mann–Whitney *U*-test; prokaryotes *P* < 0.026, virus *P* < 0.000, HNF *P* < 0.000; *n* = 34), revealing significant seasonal differences in the occurrence of these organisms.

### Prokaryotic abundance, morphotype, and biomass

**Figure 1:**
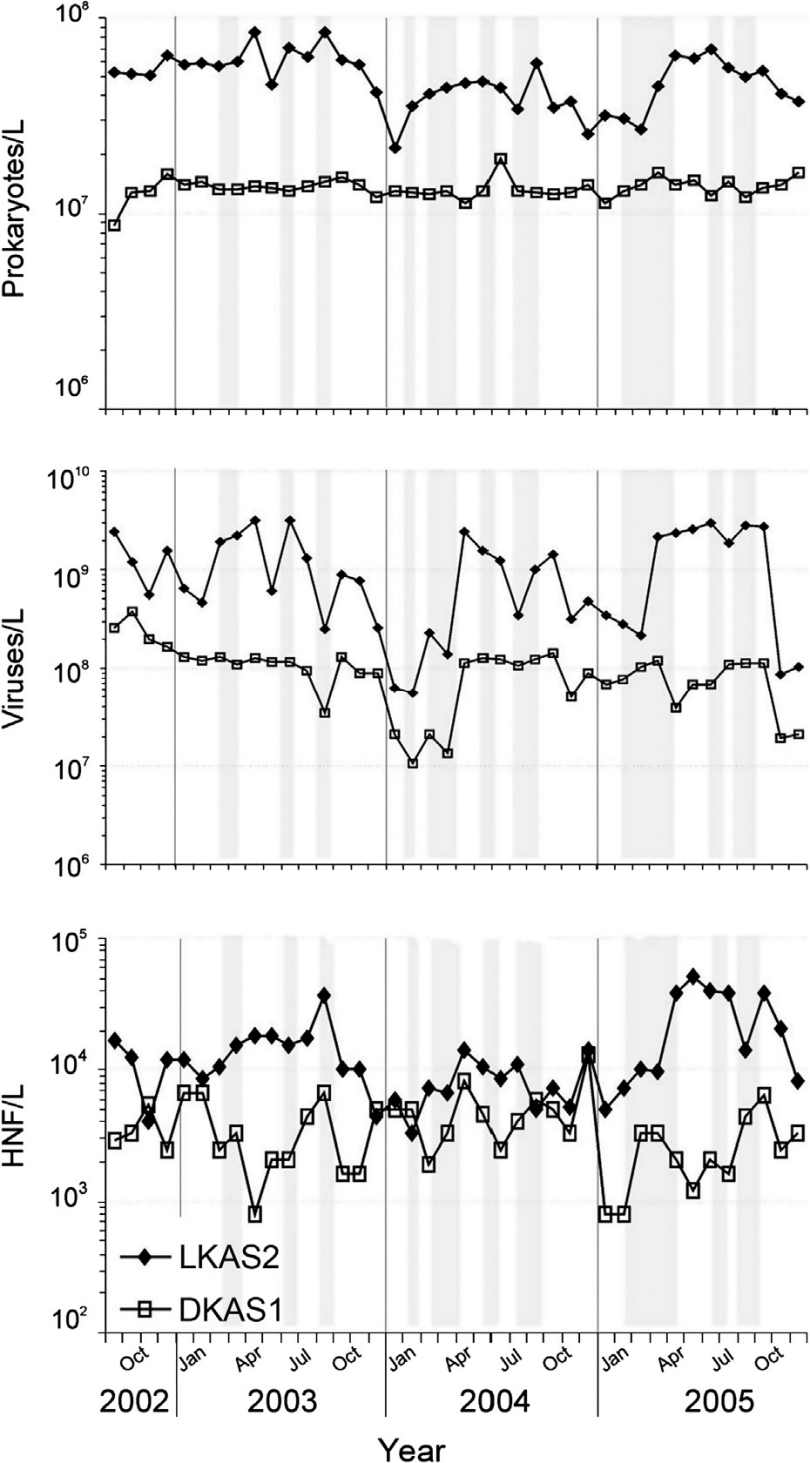
Abundance and variation of viruses, HNF, and prokaryotes in groundwater from two different alpine karst springs (DKAS1 [□], LKAS2 [♦]) monitored during three annual cycles. Gray bars indicate flood events, with discharge rising above 4500 L sec^−1^. Orthographic lines mark the beginning of each year.

Prokaryotic abundance for both springs were in the range of 10^6^–10^7^ cells L^−1^ during the whole investigation period (Table 1). Cell counts from DKAS1 were always lower than in LKAS2 showing almost no seasonal variation. The coefficient of variation for cell counts in DKAS1 was 13% in comparison to 29% in LKAS2. The more pronounced fluctuations of prokaryotic numbers (PN) in LKAS2 were correlated to discharge (*r* = 0.68, *P* ≤ 0.01, *n* = 40) (Table 2). Thus, these variations reflected the seasonal, hydrological changes showing an increase during flood events, and a constant decrease in the winter months (Fig. 1), supported by correlations with SAC_254_ and HPC22 (Table 2A). Rods and coccus-shaped cells generally comprised ≥90% of the prokaryotic community in both spring habitats (Table 1). In LKAS2 the abundance of rods clearly dominated over coccus-shaped cell numbers (Table 1), although this ratio clearly decreased in winter. The ratio in DKAS1 appeared more or less balanced throughout the year (Table 1). The observed PB for DKAS1 and LKAS2 was in the range of 8–16 nmol C L^−1^ and 32–106 nmol C L^−1^, respectively (Table 1), and accounted for about 73% of total biomass in both springs. Prokaryotic abundance obtained by FCM and EFM followed the same pattern throughout the whole year. Concerning prokaryotes, the values obtained in DKAS1 were similar and averaged to 1.4 × 10^7^ for both EFM and FCM with an averaged standard deviation of 2.9 × 10^6^ L^−1^ between the two methods. The same was true for base flow conditions in LKAS2 (discharge <4500 L sec^−1^). However, during rainfall events absolute numbers diverged, with FCM counts being higher. As a result, averaged PNs in LKAS2 were 4.4 × 10^7^ L^−1^ for EFM and 7.8 × 10^7^ L^−1^ for FCM with an averaged standard deviation of 2.6 × 10^7^ L^−1^ between the two methods.

### Viral abundance and biomass

Viral abundance determined via EFM ranged from 1.1 × 10^7^ to 3.8 × 10^8^ L^−1^ (average 1.1 × 10^8^ L^−1^) in DKAS1 and varied over two orders of magnitude in LKAS2 from 5.5 × 10^7^ to 3.2 × 10^9^ L^−1^ (average 9.4 × 10^8^ L^−1^) (Table 1, Fig. 1). Viral abundance determined by FCM was higher in both springs in comparison to microscopic analysis, ranging from 1.9 × 10^8^ to 6.4 × 10^8^ L^−1^ in DKAS1 (average 4.2 × 10^8^ L^−1^) and from 3.2 × 10^8^ to 5.4 × 10^9^ L^−1^ in LKAS2 (average 1.4 × 10^9^ L^−1^). However, the temporal trend of viral abundance over one seasonal cycle was the same. For LKAS2 there was a high correlation between EFM and FCM results for base flow conditions (*r*^2^ = 0.95; *n* = 14). During flood events FCM counts were higher than EFM counts and a weaker correlation (*r*^2^ = 0.83; *n* = 12) was observed than for base flow conditions. Then again, the standard deviation of replicates during flood events increased as well and was higher than the difference between EFM and FCM counts. The relative fraction of viral biomass of total biomass in LKAS2 was almost twice as high as in DKAS1, namely 18% and 10%, accounting for an average of 16 nmol C L^−1^ and 2 nmol C L^−1^, respectively (Table 1). Irrespective of the method used, total PNs and viral numbers did not show significant correlation in both springs. However, there was a highly significant correlation between rods and viruses (ρ = 0.68, *P* ≤ 0.01, *n* = 40) (Table 2A) in LKAS2.

Based on the EFM-data, the median virus-to-prokaryote ratio (VPR) in DKAS1 and LKAS2 was 8 (range 3–12) and 20 (range 8–43), respectively. During base flow the VPR in LKAS2 averaged 10 and was therefore comparable with DKAS1. Irrespective of the method used, median VPR during one seasonal cycle was 20 for LKAS2 and 30 during rainfall events in LKAS2. However, VPR for DKAS1 was higher (VPR = 27) when calculated from the FCM data set.

### HNF abundance and biomass

Mean HNF abundance for DKAS1 and LKAS2 was 3.3 × 10^3^ cells L^−1^ and 10.9 × 10^3^ cells L^−1^, respectively (Table 1). The highest HNF values were observed during increased discharge conditions (Fig. 1). The flagellates were dominated by small cells ranging between 2 and 5 μm (mean 3.5 μm) in cell length. Larger cells were only observed occasionally during flood events in LKAS2, but their contribution to the HNF assemblage was very small. In LKAS2 there was a significant correlation between the numbers of prokaryotes and HNF (*r*^2^ = 0.61, *P* ≤ 0.05, *n* = 40) and a highly significant correlation between HNF and viruses (*r*^2^ = 0.67, *P* ≤ 0.01, *n* = 40). HNF biomass in LKAS2 averaged 8.9 nmol C L^−1^ (Table 1). In DKAS1 HNF biomass was lower averaging 2.7 nmol C L^−1^ and PB was correlated to the numbers of HNF (*r*^2^ = 0.614, *P* ≤ 0.05, *n* = 40). The relative biomass of HNF was comparable in both spring types and accounted for 10% and 13% of total biomass in LKAS2 and DKAS1, respectively.

## Discussion

Despite the growing awareness of the importance of groundwater microbes for providing ecosystem functions (Gibert and Culver 2009) the focus within the field of groundwater microbiology is still on the production of drinking water and degradation of contaminants (e.g., Husman et al. 2009). Hitherto, there is no clear idea on interactions between the natural microbial communities, carbon turnover rates, or food web structure. The aim of this study was to investigate alpine karstic spring water from a dolomitic spring type with high average water residence time (DKAS1) and a dynamic limestone spring type that is susceptible to surface influence (LKAS2), for their naturally occurring prokaryotic, viral, and HNF abundance and to give a first descriptive insight into their dynamics during a 3-year study. The chosen sampling dates covered the full range of discharge variability, which revealed two distinct aquifers. These trends are mirrored in viral and protozoan abundance and suggest different regulation patterns of the microbial community depending on the aquifer type.

### Dependence on the hydrological situation

In accordance with the uniform discharge conditions in DKAS1, biological parameters showed low seasonal variation. The prokaryote data indicate a high biostability in older water masses throughout the year, which is in accordance with an earlier study were heterotrophic prokaryotic production and DOC was measured in the same two aquifers (Wilhartitz et al. 2009). However, there was a decrease in viral numbers during winter, which can hardly be explained by measured hydrological parameters. In LKAS2, conductivity, as an indicator of mass transport, indicated an enormous increase of suspended particles in response to rainfall or snow break events (Table 2A). The concomitantly observed higher prokaryotic and viral abundance during increased discharge can be explained by (1) detachment of cells from biofilms like in streams and rivers (Blenkinsopp and Lock 1994) and (2) an increased input of allochthonous cells and particles by surface runoff (Pronk et al. 2009b) providing (3) enhanced substrate supply, which possibly stimulates the growth of the indigenous prokaryotes. Especially the input of allochthonous cells is possibly also responsible for the strong correlation between PNs and the standard microbiological parameter HPC22, as the amount of culturable cells is extremely low during base flow conditions. Furthermore, the obvious dependence of the presence of *E. coli* and HPC22 on surface runoff suggests that microbiological water quality in LKAS2 is rather high during base flow conditions and decreases massively during situations with enhanced discharge. Significant correlations with temperature (e.g., HPC, *E. coli*) in LKAS2 (Table 2A) can be ascribed to surface runoff that is usually characterized by higher temperatures than the groundwater itself. The periodical decrease of PNs during winter is caused by the snow cover on the mountain, preventing strong surface runoff (Farnleitner et al. 2005).

### Natural viruses in alpine karst groundwater

The abundance of natural viruses in the present study was low compared to other systems (Wommack and Colwell 2000) but comparable with a recent study carried out in another aquifer type (Roudnew et al. 2013). Viral numbers showed a high correlation with discharge in LKAS2. However, there was only a marginal coupling between prokaryotes and viruses in LKAS2 during enhanced surface input indicating that during these periods, prokaryotic and viral abundances could be governed by another factor and are not necessarily linked to each other. It is important to consider, that the decrease in PNs during the winter period in LKAS2 is mainly caused by a decrease in rod-shaped cells (Farnleitner et al. 2005; Wilhartitz et al. 2009). This is most likely caused by nutrient limitation as most authors agree that starvation results in a reduced cell size caused by alterations of cell properties (Kjelleberg 1993; Morita 1997). Noteworthy, there was a significant correlation between viruses and the larger, rod-shaped cells throughout the year (*r*^2^ = 0.68, *P* ≤ 0.01, *n* = 40); Table 2), whereas no significant correlation between viruses and total prokaryotic abundance was detected. This correlation may be the result of HNA (high nucleic acid) cells representing the major source of viral production as has been suggested for other aquatic systems (Bouvier and Maurice 2011).

Generally, the average fraction of viral biomass of total biomass was considerably lower in DKAS1, namely 10% versus 18% in LKAS2. These findings are in line with evidence for higher occurrence of lysogeny and thus a lower number of free viruses in environments with lower bacterial and primary production and reduced prokaryotic abundance (Weinbauer et al. 2003; Deng et al. 2012).

Similarly, in DKAS1 the spring effluent during winter possibly contains a bigger fraction of older water masses that are depleted in nutrients, which would explain the lower abundance of free viruses during that time although discharge is stable.

The VPR has been shown to be higher for limnetic systems (around 20) than for marine pelagic systems (around 10), a difference that could be ascribed to the increased dependence of freshwater bacteria on allochthonous material (Weinbauer and Rassoulzadegan 2004). Due to the pronounced seasonal variation in VPR in LKAS2 a seasonal differentiation between base and high flow periods seemed advisable. The significantly different VPR, averaging 32 during high flow conditions and 13 during base flow, were in accordance with changes in nutrient availability. In contrast, the averaged VPR in DKAS1 was 8 (SD ± 3) during the 3 year period.

### HNF

The results of this long-term study show that HNF abundances, compared to other systems, were very low in both springs (Gasol 1994; Simek et al. 2001; Vazquez-Dominguez et al. 2008). Therefore, conclusions about their particular role and their interaction with the planktonic prokaryotic community, based on the obtained correlations, have to be treated with care.

An aspect that has to be considered is that HNF can be subdivided into three broad categories based on swimming behavior and movement: creeping flagellates mostly associated with soil surface, flagellates swimming actively in the pore water and flagellates which cannot only swim but are also capable of temporary attachment to solid surfaces (Novarino et al. 1997). It has been suggested that protist populations living in benthic environments have a wide range of feeding strategies in order to maximize niche segregation, including grazing preferences for attached/benthic and aggregate bacteria (Sibbald and Albright 1988; Starink et al. 1994). The comparatively high prokaryotes-to-heterotrophic nanoflagellates ratio found in this study, namely 4721 for LKAS2 and 5353 for DKAS1, potentially indicate a selection against planktonic prokaryotes and an uncoupling between prokaryotes and HNF in the water column (Vazquez-Dominguez et al. 2005; Baltar et al. 2012). One possible explanation may be the metabolic state of the planktonic prokaryotes, as microautoradiography results in these aquifers showed that only <10% of these cells take up [^3^H]leucine, whereas heterotrophic prokaryotic production increased by a factor of 10^6^, when measured in the aquifer sediment (Wilhartitz et al. 2009). Thus, the attached communities are probably a better energy source than planktonic cells. Selective grazing by protists on the prokaryotes which are most frequently dividing has already been observed in a marine environment (Sherr et al. 1992).

**Figure 2:**
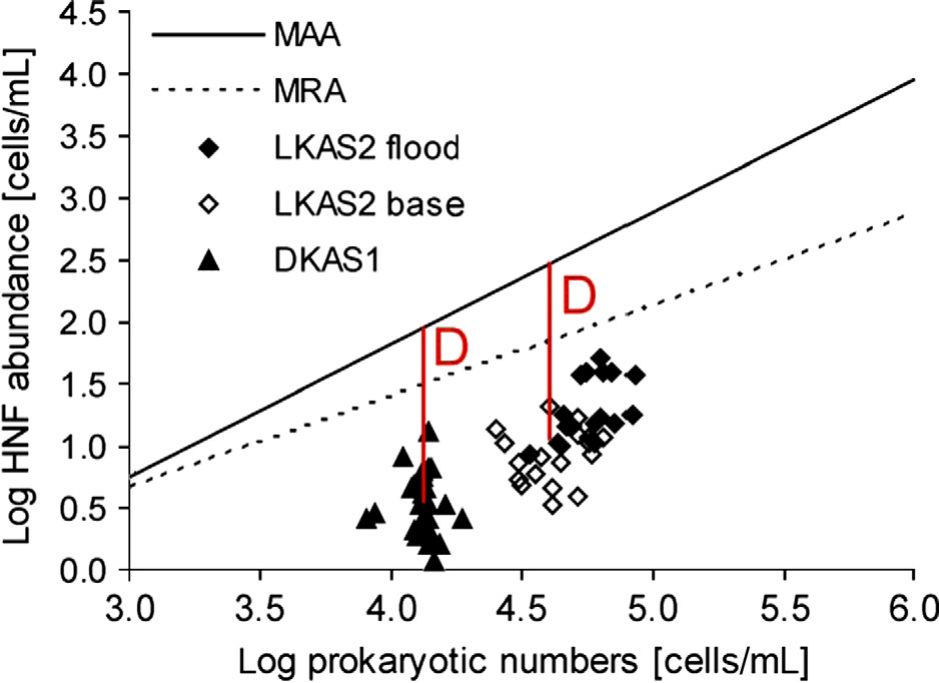
Simultaneous observation of prokaryotic and HNF abundances in groundwater from two different alpine karst springs. DKAS1 (▲) and LKAS2 during base flow (♢) and flood events (□). Data were integrated into the model proposed by Gasol (1994). MAA (–) depicts the “maximum attainable abundance” line referring to HNF, and MRA (--) the “mean realized abundance” line as they were calculated by Gasol. Points close to the MAA line indicate bottom-up control of HNF abundance, while points below the MRA line would suggest predatory control of HNF. D (red line) as the degree of uncoupling between prokaryotes and their predators was described as the distance between the maximal and the realized HNF abundance at each prokaryotic concentration level.

With regard to the low numbers of HNF, a comparison with other systems seemed advisable. Gasol (1994) proposed a qualitative model to estimate the main mode of regulation of HNF abundance in a given system where HNF and PN data are available, that was since then also used in other studies (e.g., Vazquez-Dominguez et al. 2008). The data sets of LKAS2 and DKAS1 were plotted in such a graph. Gasol applied the *D*-value (distance to the MAA-line [maximum attainable abundance]) as a surrogate for the grazing pressure of HNF on bacteria (Gasol 1994; Gasol et al. 2002). The calculated *D*-value for both springs was rather high compared to values used in the study mentioned (DKAS1: 1.52; LKAS2: 1.47) indicating a high degree of uncoupling between these two microbial compartments. Furthermore, the same model clearly showed, that all data points lie below the “mean realized abundance” (MRA) line (Fig. 2). The distance from the MRA line suggested a top-down control of HNF by zooplankton throughout the year in both spring types. A variety of different taxa, for example, Copepoda or Amphioda, have already been described in subterranean ecosystems (Galassi et al. 2009; Gibert and Culver 2009; Gibert et al. 2009), including reports about karst-dwelling planktonic Calanoida (Brancelj and Dumont 2007).

The results would imply that prokaryotic abundance in the water column is not controlled by HNF predation as was suggested for other oligotrophic open water systems (Gasol et al. 2002). In this context the correlation between HNF and prokaryote numbers observed in LKAS2 could be ascribed to an indirect coupling due to changing water levels increasing the shear stress in the system and causing the detachment of HNF from the biofilm as well as direct surface input of allochthonous organisms.

A crucial factor to consider in this kind of environment is the biofilm compartment. The ratios between prokarotes, viruses, and HNF found in this study for the planktonic fraction probably differ considerably from the attached compartment; as was already seen for prokaryotes (Wilhartitz et al. 2009). Therefore, some contradictory results with existing models for aquatic environments could be explained by the fact that karstic groundwater in itself should not be seen as self-contained planktonic habitat, but as one compartment of a bivariate environment.

## Conclusion

Ultraoligotrophic groundwater from two distinct alpine karst springs has been investigated for their natural prokaryotic, viral, and heterotrophic nanoflagellate abundances during three seasonal cycles. The study revealed basic differences between the aquifers, depending on the hydrological situation. Generally, the virus-to-prokaryotes ratio was in the range of values found in other aquatic environments suggesting that lysogeny prevails in older water masses. However, the prokaryotes-to-heterotrophic nanoflagellates ratio in the planktonic fraction was unambiguously higher than described for other systems, irrespectively of the hydrological situation. The low grazing pressure on planktonic prokaryotes implies that (i) their abundance is mainly controlled by availability of nutrients and death due to viral lyses and (ii) the grazing pressure on allochthonous prokaryotes, including possible pathogens, in the water column is negligible.
